# Integrating a Chemicals Perspective into the Global Plastic Treaty

**DOI:** 10.1021/acs.estlett.2c00763

**Published:** 2022-11-22

**Authors:** Zhanyun Wang, Antonia Praetorius

**Affiliations:** † Empa − Swiss Federal Laboratories for Materials Science and Technology, Technology and Society Laboratory, 9014 St. Gallen, Switzerland; ‡ Institute for Biodiversity and Ecosystem Dynamics, University of Amsterdam, Amsterdam 1090, GE, Netherlands

**Keywords:** global plastic treaty, nonintentionally added substances (NIAS), plastic additives, plastic processing aids, plastic recycling, waste-to-energy, biobased plastics, biodegradable plastics, durable plastics

## Abstract

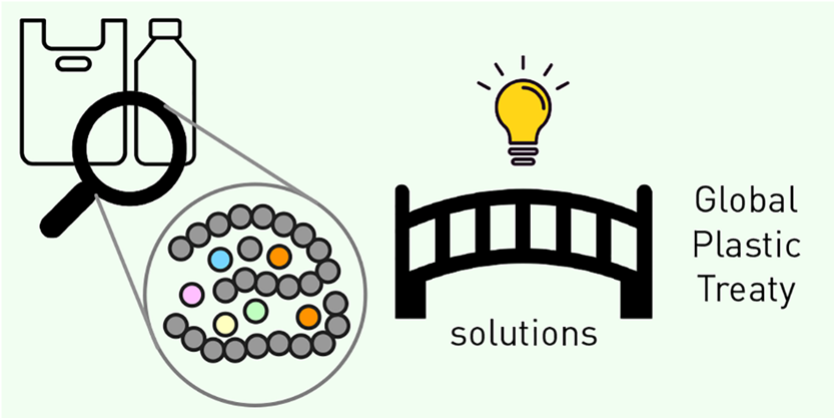

Driven by the growing concern about plastic pollution, countries have agreed to establish a global plastic treaty addressing the full life cycle of plastics. However, while plastics are complex materials consisting of mixtures of chemicals such as additives, processing aids, and nonintentionally added substances, it is at risk that the chemical aspects of plastics may be overlooked in the forthcoming treaty. This is highly concerning because a large variety of over 10,000 chemical substances may have been used in plastic production, and many of them are known to be hazardous to human health and the environment. In this Global Perspective, we further highlight an additional, generally overlooked, but critical aspect that many chemicals in plastics hamper the technological solutions envisioned to solve some of the major plastic issues: mechanical recycling, waste-to-energy, chemical recycling, biobased plastics, biodegradable plastics, and durable plastics. Building on existing success stories, we outline three concrete recommendations on how the chemical aspects can be integrated into the global plastic treaty to ensure its effectiveness: (1) reducing the complexity of chemicals in plastics, (2) ensuring the transparency of chemicals in plastics, and (3) aligning the right incentives for a systematic transition.

Plastics have become ubiquitous in modern life thanks to their characteristics such as being lightweight, low-cost, and versatile. As a result, global plastic production has increased drastically in the past decades, reaching about 370 megatonnes in 2020, with continued growth expected and possibly reaching 1.3 gigatonnes in 2060.^1^ Meanwhile, the world has also witnessed a tremendous increase of plastic waste in the environment, which has led to many associated adverse impacts on human health and the environment.^1−7^ Driven by the growing public and scientific concerns, at the United Nations Environment Assembly meeting (UNEA 5.2) in March 2022, countries agreed to establish a global treaty on plastic pollution by 2024 to address the full life cycle of plastics.^8^ This is a key milestone toward addressing global plastic pollution; however, the many twists and turns in the negotiations during UNEA 5.2 suggest the forthcoming treaty negotiations being anything but easy, starting from defining the treaty scope.^9^ We echo Simon et al.^10^ that the treaty needs to address hazardous chemical additives to facilitate safe circularity of plastics, which has been included in the official meeting documents that are to facilitate deliberations at the first treaty negotiation meeting in November 2022.^11,12^ This is encouraging, yet not sufficient.

Plastics are complex materials, composed of mixtures of diverse components.^13^ The bulk component is an organic polymer matrix made from repeating synthetic monomer units. Depending on the polymer matrix, plastics can be grouped into thermoplastics (e.g., polyethylene, polypropylene; ca. 90% of the plastics) or thermosets (e.g., epoxy, polyurethane; ca. 10% of the plastics).^14^ Further, various chemicals are added in plastic production, as processing aids to enable or ease the production and processing of the plastics (e.g., lubricants) or as additives to maintain, enhance, and impart specific properties (e.g., plasticizers, flame retardants, antioxidants). A recent study has identified more than 10,000 chemical substances that may have been used as monomers, processing aids, and additives.^13^ Thus, plastics may contain a wide range of chemical substances that are not chemically bound to the polymer matrix, including both intentional ones, such as unreacted monomers, residual processing aids, and additives,^13^ and nonintentionally added ones, such as reaction byproducts, breakdown products, and contaminants from the raw materials and production processes.^15,16^

Note that a wide range of chemicals may fulfill the same function.^13^ This, in combination with the general lack of coordination among manufacturers, has resulted in plastics from different manufacturers for the same applications—even with the same polymer matrix—often having varied chemical compositions.^17^ Such diversity and complexity of chemicals in plastics can cause various negative impacts and challenges. Particularly, the release and (potential) adverse human and ecosystem health effects of many intentionally added chemicals (e.g., many bisphenols, phthalates, alkylphenols, toxic metals, and flame retardants) and nonintentionally added chemicals have been widely studied, recognized, and raised as major concerns.^13,15,16,18−20^ Equally important but often overlooked is the fact that diverse chemicals in plastics can pose many challenges to the current and envisioned technological solutions to plastic pollution.

We strongly recommend that chemicals present in plastics should be an integral part, in order for the treaty to be effective.^11,12^ Here, we call for a broader and more comprehensive consideration of chemicals in plastics beyond solely hazardous ones, including all intentionally and nonintentionally added chemicals that are not chemically bound to the polymer matrix. In particular, we highlight the often-overlooked aspect that many chemicals in plastics can impede existing or proposed technological solutions for addressing plastic pollution, even without being hazardous to human health and the environment according to the current state of the science. Then, building on existing success stories, we recommend three key elements to be considered for integration in the treaty to address the chemical aspects of plastics in a holistic manner.

## Chemicals in Plastics Inevitably Influence the Effectiveness of Technical Solutions

Here, we take a closer look at the impacts of chemicals in plastics on the feasibility and effectiveness of the major existing and proposed technological solutions for addressing plastic pollution. Note that the following subsections are not intended to be an exhaustive review of all challenges, but to highlight common challenges associated with chemicals in plastics using some common examples; more details of individual examples can be found in the Supporting Information. Further challenges may also be present, e.g., associated with high energy consumption or land-use change, but are not discussed here.

### Mechanical Recycling

Mechanical recycling has been widely deployed for many thermoplastics, but not for thermosets as the polymer matrix breaks down at high temperature during recycling.^21^ The processes of mechanical recycling affect the makeup of chemicals in the plastics, as they may be retained, transformed, and/or released. The presence of certain intentionally added chemicals and their transformation products can reduce the marketability of secondary plastics in multiple ways: Pro-oxidant additives that stimulate oxidation may break down the polymer matrix during the recycling processes and, thus, lower the technical quality of recyclates.^22,23^ Formation of dark color may occur from mixing different additives/pigments, reducing the aesthetics of secondary plastics.^24−26^ Formation of toxic byproducts may occur such as brominated dioxins and furans (PBDD/Fs) from brominated flame retardant precursors at unsuitable temperatures used during recycling processes^27^ (Supporting Information, Sections S1.1–S1.3). In addition, the presence of diverse additives can reduce the compatibility of different waste streams, even with the same polymer type, as seen in the example of polyethylene terephthalate (PET) where PET bottles and trays cannot be mixed during recycling (Section S1.4).^17^ This is because the chemical compositions of PET trays are more diverse in comparison to PET bottles; during the grinding steps of the recycling process, PET trays tend to produce smaller scraps, more heterogeneous parts, and more dust which might not be efficiently recycled.^17^ Furthermore, concern about contamination of secondary plastics by legacy hazardous additives in input waste plastics has resulted in some parts of the world limiting certain waste plastics from recycling (e.g., in the European Union, waste plastic containing persistent organic pollutants (POPs)^28^ or other restricted brominated flame retardants,^29^Section S1.5). In other parts where such plastics are still recycled, exposure of workers and nearby communities to hazardous additives released during recycling has been well documented (Section S1.6).^30−32^

### Waste-to-Energy

Two major types of processes exist, generating electricity and/or heat from plastic incineration or processing of waste plastics into a fuel source through pyrolysis. The former can result in large amounts of exhaust and bottom ashes containing significant levels of microplastics,^33^ as well as hazardous additives and their thermal transformation products (e.g., toxic metals,^33^ dioxins^34,35^), which require additional safe handling measures (Section S2.1). For waste-to-fuel, in addition to contaminated exhaust and residues for incineration, the presence of many metals and halogenated chemicals may impact yields, result in lower quality of the end fuel products, and pose technical problems to the thermal facilities and equipment using the fuels (Section S2.2).^36−39^

### Chemical Recycling

Here, chemical recycling refers to those processes such as gasification and depolymerization that alter the plastic’s chemical structure to produce substances that can be used as products (excluding fuels) or as raw materials for the manufacturing of new polymers. High-temperature processes, e.g., gasification and using pyrolysis oil as a steam cracker feedstock, face similar challenges as stated above for waste-to-energy (Section S3.1).^37,40−42^ The presence of certain chemical additives can result in unintended side reactions during chemical recycling and, thus, lower the overall efficiency and increase the generation of process waste that would require safe handling measures (Sections S2.2 and S3.1). Thus, similar to mechanical recycling, chemicals in plastics may lead to incompatibility of different waste streams, even of the same polymer type, as feedstocks for chemical recycling.

### Biobased Plastics

Biobased commodity plastics such as biopolyethylene (bio-PE) produced from biomass feedstocks can contain the same range of chemical additives as their fossil-based counterparts (Section S4.1),^43,44^ as the use of additives depends on the processing steps and desired product properties rather than on the original feedstock. For example, to enhance the durability and plasticity of otherwise brittle and rigid biobased plastics produced from polylactic acid (PLA), conventional additives, such as fillers and/or plasticizers, are needed (Section S4.2).^45,46^ Furthermore, the production of food-contact plastics based on high levels of plant fibers (e.g., bamboo plastics) involves the use of several additives that may pose high risks to food safety (e.g., melamine) (Section S4.3).^47,48^

### Biodegradable Plastics

Biodegradation is highly dependent on material composition and external factors. Most fossil-based or biobased biodegradable plastics only truly mineralize in industrial composting facilities, but only partially (or very slowly) disintegrate in natural environments.^49^ Depending on the specific additives and polymer composition of the biodegradable plastic, as well as the environmental conditions, environmental disintegration of littered biodegradable plastics may not only result in the formation of microplastics or nanoplastics, but also facilitates the release of chemical additives (Section S5.1).^50−52^ In addition to broader ecosystem impacts, the released additives may impede the biodegradation process itself if they are harmful to the degrading microorganisms.

### Durable Plastics

Increasing durabilities of plastic products has been called for to prolong product lifetimes and enable multiple reuse cycles, as a means to help curb overall production of new plastics. For such durable products to be feasible, the lifetimes of all embedded chemical additives need to align with the anticipated/desired plastic lifetime, including expected use and cleaning conditions. For example, durable plastics and their additives may need to withstand multiple (dish)washing cycles without (or with only minimal) leaching and/or transformation of embedded additives, or absorption of additional chemicals (Section 6.1).^53^ Additive migration during the intended use phase can accelerate the failure of plastic products and impede intended long-term use, e.g., due to product embrittlement or lack of the function initially provided by the additive (Section S6.2).^54^ Some additives may specifically contribute to increasing plastic longevity (e.g., photostabilizers), but sometimes, the protective function generates unintended negative impacts. A prominent example is a tire rubber additive—*N*-(1,3-dimethylbutyl)-*N*′-phenyl-*p*-phenylenediamine (6PPD), which upon deploying its protective antioxidative function, yields hazardous transformation products including 6PPD-quinone, a chemical that is now known to be highly toxic to many aquatic species (Section S6.3).^55−57^

## Recommended Key Elements for Addressing Chemicals in Plastics in a Holistic Manner

Challenges posed by the multifaceted impacts of chemicals in plastics are heightened by the fact that >10,000 substances may have been used in the plastic production and processing, and in most cases, public information about the identities and levels of chemicals in specific plastic products is missing.^13,15,58^ Essentially, the diverse use of chemicals in plastic production and processing renders current and proposed technological solutions ineffective at best and counterproductive at worst (as they may, e.g., distract policymakers and the public from real solutions).

To solve the growing plastic waste issue, concerted action on identifying, understanding, and sound management of chemicals in plastics is critical and even a prerequisite. Attempts, both voluntary and legally binding, have been made to address chemicals in plastics,^59−61^ but generally in a fragmented and narrowly scoped manner, limited to specific chemicals, products, industrial sectors, and jurisdictions (e.g., phase-out of bisphenol A in polycarbonate baby bottles in some parts of the world). We recommend the treaty as a global platform to systematically and comprehensively tackle the matter, with the following elements for consideration.

It should be noted that this notion of addressing chemicals in plastics does not preclude any action on restraining plastic consumption and production,^62,63^ particularly single-use ones, given the worldwide limited technical capacity to handle plastic waste^64,65^ and their already ubiquitous presence in the environment.^2^ Rather, restraining the consumption and production allows us to focus the finite human and financial resources on better addressing chemicals in those plastics that are currently in uses essential for the health, safety, and functioning of our society but without technically and economically feasible alternatives that are acceptable from the standpoint of the environment and health.^58^

### Reducing the Complexity of Chemicals in Plastics

This is not only beneficial and much needed,^66−68^ but also practically feasible. A success story for reducing chemical complexity and harmonizing plastic formulations is the PET bottle-to-bottle recycling. While challenges remain related to the physical limits of mechanical recycling,^67^ the urgent need to minimize/eliminate nonessential use of single-use plastics,^10^ and the insufficiently understood fate of nonintentionally added chemicals during multiple cycles of recycling,^69^ the PET bottle example is a clear step in the right direction. In this case, industry actors from the whole value chain were convened and agreed on the “Design for Recycling Guidelines” that streamline and standardize the use of additives, and thus ensure the recyclability of PET bottles from different manufacturers and their compatibility for mixing before recycling.^70^

The plastic treaty can learn from the PET-bottle case and expand it to other use areas. For example, in accordance with the UNEA 5.2 outcomes,^8^ the treaty can establish a global forum to convene actors and experts along the entire value chain of certain use applications (including brand owners; manufacturers of polymers, additives and machinery; recyclers). The forum would be mandated to evaluate and agree on streamlined and standardized recipes of the plastics, including phase-out of known hazardous chemicals, and to update them as knowledge advances. Such recipes need to include guidance on how to select or design chemical additives for needed functions and properties, but with simplicity, safety, end-of-life treatability, and compatibility across waste streams in mind. Ideally, a well-defined set of safe additives, which serve certain key functions and properties, can be identified, drastically reducing the diversity and complexity of chemicals in plastic products. Focusing on a much smaller set of chemicals and harmonized product formulations will also make it substantially more feasible to assess potential hazards of the chemicals used in plastic production and ensure product safety. The idea is not to limit innovation, but to guide innovation toward achieving more with fewer, well-understood chemicals (e.g., via multifunctional additives).

The forum needs clear targets and periodic evaluation to ensure its effectiveness (e.g., the treaty can start with a voluntary forum with predefined targets, and then possibly introduce legally binding regulations if the targets are not met, similar to the settings in the Swiss Ordinance on Beverage Containers^71^). As plastics are used in virtually every possible area of our society and each area has its own needs, the forum may need to prioritize its activities. For example, it may first focus on those areas with high production volumes, high environmental release and/or human exposure potential, and a manageable size of key actors. The future global science-policy panel on chemicals, waste, and pollution prevention^72^ can, for example, be tasked to assess and identify key areas to be prioritized.

In parallel to the forum, the treaty may establish a formal process for timely identification and management of certain hazardous chemicals in plastics that warrant international action but cannot be addressed by existing multilateral agreements. Such a process can be adapted from, e.g., the Stockholm Convention on Persistent Organic Pollutants.^73^

### Ensuring Transparency on Chemicals in Plastics

Another central missing piece is information on the identities and amounts of chemicals present in the multitude of plastics currently in use. Such information gaps do not only exist in the public domain, limiting the public’s right to know and oversight, but also along many supply chains, limiting the ability of environmentally conscious businesses down in the supply chain to take informed action. Therefore, a publicly accessible database listing all intentionally added chemicals per plastic product needs to be implemented by the industry when placing a product on the market. The global automobile industry has demonstrated the feasibility of such an approach through its International Material Data System (https://www.mdsystem.com), where all materials used are collected, maintained, analyzed, and archived (although currently only accessible to the supply chain actors). Such efforts need to be upscaled to cover other industrial sectors and to make information also accessible to the public.^58^ To balance public access and protection of trade secrets for businesses’ comparative advantages, the public disclosure of ingredients in cleaning products may be used as an initial model,^74^ where the chemical identities are listed in a sequence following their levels in the products without providing the exact numbers.

One particular challenge is posed by nonintentionally added substances (NIASs),^15^ where a true lack of knowledge on the identity of reaction byproducts, breakdown products, and contaminants is the bottleneck. An increased mechanistic understanding of the sources and formation of NIASs is needed to optimize plastic production processes to minimize them. Here, close collaborations between scientists and the industry are essential. Additionally, suspect and nontargeted chemical analytical approaches need to be further developed and applied to systematically identify and monitor both intentionally added chemicals and NIASs in plastics and the environment. A starting point can be those plastics with high production volumes or high exposure potential. Then, the treaty can include (or encourage the inclusion of) chemicals explicitly in global monitoring schemes, such as the recently proposed Global Plastic Pollution Observatory System by scientists,^75^ to identify and prioritize both plastics and associated chemicals in environmental systems.

### Aligning the Right Incentives for Transition

Learning from the carbon “lock-in,” in order to break the status quo and foster transitions, relevant social, economic, and political dynamics need to be properly addressed in addition to technological solutions.^76−78^ The plastic treaty offers a unique opportunity to align the current incentive systems to foster true innovation and transitions in the aforementioned areas and the general sound management of plastics **and** chemicals therein. A core consideration is to create level playing fields that can incentivize pioneers and frontrunners and encourage others to follow their paths, instead of the other way around as is often occurring now.

As economic incentives, levies can be established to promote information transparency of chemicals used in plastics, by subjecting producers to fees based on their level of intransparency and exempting those demonstrating full transparency of chemical compositions. Collected fees can be used to fund an information system for public disclosure of chemicals in plastic products. Similar levies may also be established for reducing chemical complexity in plastics. For example, adding certain types of chemicals (e.g., additives other than those agreed in the streamlined and standardized recipes; chemicals identified under the treaty) may trigger extra taxes, as Denmark previously implemented for phthalates in polyvinyl chloride (PVC).^79^ Collected fees may be used as grants for supporting small- and medium-sized enterprises in transitioning. Establishing levies at the international level may be challenging, but the plastic treaty can help to develop models that can be adopted by individual countries/regions as ways of implementing the treaty and its obligations. Additionally, fast-track regulatory approvals can be used as another mechanism to create economic incentives for truly innovative products that meet specific criteria, e.g., on transparency, simplicity, safety, and/or sustainability. Standardized certification schemes may be further established under the treaty to create social and economic incentives (e.g., branding) for such products. Additional lessons may be learned from voluntary and legally binding instruments that have been used to address single-use plastics.^80^

Furthermore, the current time lag between scientific identification of hazardous chemicals and regulatory action—up to decades long—needs to be drastically shortened, and regulations need to be stricter. This would, for instance, include accelerating the restriction of hazardous chemicals in new products as well as in products already on the market. Businesses should be required to provide a clear substitution plan for approval of continued use (of hazardous additives and/or of nonstandardized product formulations) instead of getting a blanket long transition time. This strategy can provide additional incentives for businesses to proactively streamline their use of chemicals in plastics and select additives toward Safe and Sustainable by Design.^81^ We are hopeful that the forthcoming global science-policy panel on chemicals, waste, and pollution prevention can play a key role to bridge the scientific and policymaking communities, particularly in shortening the time lag and assessing possible solutions.

## Key Messages

(1) Plastics are complex mixtures of diverse chemicals, including intentional ones, such as unreacted monomers, residual processing aids, and additives, and nonintentionally added ones, such as reaction byproducts, breakdown products, and contaminants from the raw materials and production processes.
(2) The diverse chemicals present in plastics can cause various negative impacts and challenges. In addition to the often-discussed impacts on human health and the environment, we highlight the wide range of challenges posed by chemicals to the technological solutions envisioned for addressing the plastic crisis, rendering these solutions ineffective at best and counterproductive at worst.
(3) In order to successfully end plastic pollution, holistic action is required to address chemicals present in plastics, including (1) reducing the complexity of chemicals in plastics, (2) ensuring the transparency of chemicals in plastics, and (3) aligning the right incentives for a systematic transition.

## Supporting Information Available

Supporting details on the impacts of chemical additives on mechanical recycling, waste-to-energy, chemical recycling, biobased plastics, biodegradable plastics, and durable plastics (PDF)
