# Supplementary material for: Integrating a Chemicals Perspective into the Global Plastic Treaty
Source: Environ Sci Technol Lett. 2022 Nov 22;9(12):1000–6. doi: 10.1021/acs.estlett.2c00763 (PMC9753957; doi:10.1021/acs.estlett.2c00763)
Supplement: Supplementary file 1 — ez2c00763_si_001.pdf [file ez2c00763_si_001.pdf]

Supporting Information

Integrating a Chemicals Perspective into the Global Plastic Treaty

Zhanyun Wang[1,*], Antonia Praetorius[2,*]

[1] Empa − Swiss Federal Laboratories for Materials Science and Technology, Technology and Society Laboratory, 9014 St. Gallen, Switzerland

[2] Institute for Biodiversity and Ecosystem Dynamics, University of Amsterdam, Amsterdam 1090 GE, Netherlands

* Correspondence to

Zhanyun Wang: Zhanyun.wang@empa.ch, Zhanyun.wang@ifu.baug.ethz.ch;

Antonia Praetorius: a.praetorius@uva.nl

Table of Contents

S1. Supporting Details on the Impacts of Chemical Additives on Mechanical Recycling S2
S2. Supporting Details on the Impacts of Chemical Additives on Waste-to-Energy S6
S3. Supporting Details on the Impacts of Chemical Additives on Chemical Recycling S11
S4. Supporting Details on the Impacts of Chemical Additives on Bio-based Plastics S13
S5. Supporting Details on the Impacts of Chemical Additives on Biodegradable Plastics S17
S6. Supporting Details on the Impacts of Chemical Additives on Durable Plastics S18

Notes:

1. The sections aim to complement the main text by providing supporting technical details and key references on the corresponding individual statements. To avoid misinterpretation, supporting technical details are often provided in the format of direct quotes.
2. The **quotations** in this document follow APA Style, including being highlighted using a different font Optima (https://apastyle.apa.org/style-grammar-guidelines/citations/quotations).

S1. Supporting Details on the Impacts of Chemical Additives on Mechanical Recycling

S1.1 Additives that stimulate oxidation may break the polymer matrix during the recycling processes, and thus, lower the technical quality of recyclates.

- European Commission. 2018. *Report from the Commission to the European Parliament and the Council on the impact of the use of oxo-degradable plastic, including oxo-degradable plastic carrier bags, on the environment.* https://eur-lex.europa.eu/legal-content/EN/TXT/?uri=CELEX%3A52018DC0035#footnote13; https://op.europa.eu/en/publication-detail/-/publication/bb3ec82e-9a9f-11e6-9bca-01aa75ed71a1/language-en/format-PDF/source-56357670

  5.2 Quality issues and marketability of recyclates

  Significant concerns exist within the recycling industry that oxo-degradable plastic negatively affects the quality of recycled plastics. Tests have demonstrated that the presence of oxo-degradable plastic in a conventional plastic recycling system can lead to poor quality recyclate. Even though it also appears possible to produce high quality recyclate, there is no certainty about the absence of negative impact of the oxo-degradable plastic on the recyclate.[13]

  Evidence suggests that the impacts of oxidising additives on recyclates can under certain circumstances be avoided with the inclusion of stabilisers. The appropriate quantity and chemistry of stabiliser would depend on the concentration and nature of the oxidising additives in the feedstock. However, as the concentration of oxo-degradable plastic in recyclate in real world situations is unknown, it is difficult to know the correct dosing of stabilisers.

  A major issue is furthermore that it is impossible to fully control the level of aging experienced by oxo-degradable plastics during the product use phase, prior to products becoming waste and entering recycling processes.

  The existence of oxo-degradable plastic and the global nature of markets for secondary materials present risks to a more generalised use of recovered plastic in long-life products. The uncertainty of whether the recyclate may contain oxo-degradable plastic and of the degree of oxidation and degradation that might have occurred prior to recovery limits the end-use for the recyclate, having a negative impact on the price of the recyclate and on the competitive position of the plastic recycling industry.

- The findings in the aforementioned report are further supported by
  - Aldas M, Paladines A, Valle V, Pazmiño M, Quiroz F. 2018. Effect of the pro-degradant-additive plastics incorporated on the polyethylene recycling. *International Journal of Polymer Science* 2474176, https://doi.org/10.1155/2018/2474176
  - Babetto AS, Antunes MC, Bettini SHP, Bonse BC. 2020. A recycling-focused assessment of the oxidative thermomechanical degradation of HDPE melt containing pro-oxidant. *Journal of Polymers and the Environment* 28, 699–712. https://doi.org/10.1007/s10924-019-01641-6

S1.2 Formation of dark colors from mixing plastics with different additives/pigments, reducing the aesthetics of secondary plastics.

- Mixing PVC from different streams may result in secondary PVC with undesirable black / dark grey color due to reactions of different stabilizers.
  - Schiller M. 2015. *PVC Additives: Performance, Chemistry, Developments and Sustainability*. Carl Hanswer Verlag GmbH & Co. KG. ISBN: 9781569905432. https://www.hanser-elibrary.com/doi/book/10.3139/9781569905449

    > In the presence of sulfur-containing tin stabilizers, the recycling of any lead-stabilized PVC is made difficult by the reaction of lead with sulfur to form black lead sulfide. (p. 27)

  - Titow WV. 1990. Chapter 4. Stabilisers in *PVC Plastics – properties, processing and applications*. Springer Netherlands. http://doi.org/10.1007/978-94-011-3834-5

    > Although simultaneous use of sulphur-containing additives with cadmium or lead compounds (say, for example, thiotin or antimony mercaptide stabilisers in conjunction with lead or cadmium ones) is avoided in formulating practice, it can occur in the processing of PVC scrap of different or uncertain origins. Where fresh stabiliser is to be added to a batch of scrap material of unknown composition to 'post-stabilise' it for processing and service, or where two or more such batches are to be mixed in processing, the possibility of cross-staining should be checked beforehand (even for black material). (p. 155)

- The Sustainable Packaging Coalition. n.d. Design for Recycled Content Guide. https://sustainablepackaging.org/projects/design-for-recycled-content-guide/

  > Color consistency and color matching are common challenges in using recycled plastic, since brands tend to implement very stringent color requirements for packaging. Unlike virgin plastics, which initially do not contain pigments, recycled plastics are derived from mixtures of materials that may contain a wide range of pigmentation. Brands interested in using recycled plastics must manage their expectations and commit to finding ways to work with the color variations present in recycled resins.
  >
  > White or lightly colored recycled plastics may take on an off-white color. Clear recycled plastics may take on a somewhat yellowed appearance due to the reheating process, or a cloudy appearance due to contamination in the recycled feedstock. Natural, white or lightly colored recycled plastics can be adjusted by adding colorants to match brand colors, however, their new color may appear less vibrant than virgin material colored with the same colorant. Mixed-color streams of recovered plastics can typically only be recycled into dark, opague colors.
  >
  > While these challenges are more pronounced with higher levels of recycled content, there are numerous examples of plastic packaging containing upwards of 30% recycled content with no or negligible aesthetic deficiencies. That percentage can be considered a general threshold above which aesthetic challenges should be expected to be more noticeable.

S1.3 Formation of toxic by-products from chemical additives during recycling

- Budin C, Petrlik J, Strakova J, Hamm S, Beeler B, Behnisch P, Besselink H, van der Burg B, Brouwer A. 2020. Detection of high PBDD/Fs levels and dioxin-like activity in toys using a combination of GC-HRMS, rat-based and human-based DR CALUX® reporter gene assays. *Chemosphere* 251, 126579. https://doi.org/10.1016/j.chemosphere.2020.126579 (CC BY)

> Brominated dioxins are originally present as impurities in some commercial brominated flame retardant mixtures, such as decabromodiphenyl ether (DecaBDE) or other PBDE mixtures which are used to flame-retard plastics and electronics (Altarawneh et al., 2019; Buser, 1986; Ren et al., 2017, 2011). Main sources of PBDD/F formation are during incomplete thermal degradation of flame-retarded plastics. Unsuitable temperature conditions used during recycling processes may also lead to the formation of PBDD/Fs from brominated flame-retardant precursors (Ebert and Bahadir, 2003; Hamm et al., 2001; Zhan et al., 2019).

S1.4 The presence of diverse additives can reduce the compatibility of different plastic waste streams with the same polymer type.

- Mixing PET trays in the PET bottle recycling reduces the quality of recycled PET.
  - European Commission. 2019. *A circular economy for plastics – insights from research and innovation to inform policy and funding decisions.* https://op.europa.eu/en/publication-detail/-/publication/33251cf9-3b0b-11e9-8d04-01aa75ed71a1/language-en/format-PDF/source-87705298

> For example, clear PET bottles end up in the same material stream as clear PET trays, where the latter are more diverse through differences due to additives and the formation process. As a result, during the grinding steps of the recycling processes, bottles will be shredded into homogenous scraps while trays will tend to produce smaller scraps, more heterogeneous parts, and more dust which might not be efficiently recycled. (p. 115)

S1.5 Concern about contamination of secondary plastics by legacy hazardous additives has resulted in regulations in some parts of the world limiting certain waste plastics from recycling.

- Waste materials containing persistent organic pollutants such as polybrominated diphenyl ethers (PBDEs) and hexabromocyclododecane above allowed concentration limits shall not be directly re-used or recycled within the EU, according to the POP Directive (EU 2019/1021).
  - POP Directive (EU 2019/1021; https://eur-lex.europa.eu/legal-content/DE/ALL/?uri=CELEX:32019R1021), Article 7 on Waste Management

> 1. Producers and holders of waste shall undertake all reasonable efforts to avoid, where feasible, contamination of this waste with substances listed in Annex IV.
>
> 2. Notwithstanding Council Directive 96/59/EC (24), waste consisting of, containing or contaminated by any substance listed in Annex IV to this Regulation shall be disposed of or recovered, without undue delay and in accordance with Part

1 of Annex V to this Regulation, in such a way as to ensure that the POP content is destroyed or irreversibly transformed so that the remaining waste and releases do not exhibit the characteristics of POPs.

In carrying out such a disposal or recovery, any substance listed in Annex IV may be isolated from the waste, provided that this substance is subsequently disposed of in accordance with the first subparagraph.

3. Disposal or recovery operations that may lead to recovery, recycling, reclamation or re-use on their own of the substances listed in Annex IV shall be prohibited.

4. By way of derogation from paragraph 2:

(a) waste containing or contaminated by any substance listed in Annex IV may be otherwise disposed of or recovered in accordance with the relevant Union legislation, provided that the content of the listed substances in the waste is below the concentration limits specified in Annex IV (p. L169/52–53)

- European Electronics Recyclers Association. 2018. *Responsible recycling of WEEE plastics containing brominated flame retardants.* https://www.kmk.ie/custom/public/files/responsible-recycling-of-weee-plastics-containing-brominated-flame-retardants-bfrs.pdf

En standard 50625-1 on collection, logistics & treatment requirements for WEEE - part 1:
General treatment requirements takes a threshold value for total elemental Bromine - Br of 2.000 ppm for the check if there are BFR's in plastics. Many product categories do not contain any BFR's or only traces of these BFR's. The check for mixed plastics from product categories that can contain BFR is done by determining the Br content e.g. all Bromine molecules in all BFR's. For electronic products there is substantial evidence that if Br < 2000 ppm no restricted BFR's above the legal thresholds can be found. So if Br > 2000 ppm it is assumed that restricted BFR's are present and further sepa- ration by specialised plastic recycling facilities is required. In these processes the restricted BFR plastic fraction is concentrated and this fraction must be incinerated. By doing so, the restricted BFR are removed from the material cycle.

S1.6 Exposure of workers and nearby communities worldwide to legacy hazardous additives released during mechanical recycling

- Huang D, Zhou S, Hong W, Feng W, Tao L. 2013. Pollution characteristics of volatile organic compounds, polycyclic aromatic hydrocarbons and phthalate esters emitted from plastic wastes recycling granulation plants in Xingtan Town, South China. *Atmospheric Environment* 71, 327–334. https://doi.org/10.1016/j.atmosenv.2013.02.011 (with permission from Elsevier)

Both inside and outside the plants, the total concentrations of volatile monocyclic aromatic hydrocarbons (MAHs), PAHs and PAEs ranged from 2000 to 3000 µg m^{-3}, 450 to 1200 ng m^{-3}, and 200 to 1200 ng m^{H}, respectively. Their concentration levels inside

the plants were higher than those outside the plants, and PAHs and PAEs were mainly distributed in the gas-phase. Notably, highly toxic benzo[a]pyrene (BaP) could be detected inside the plants, and harmful PAEs could be detected not only inside but also outside the plants, although PAEs are non-volatile. The exhaust gas composition and concentration were related to the plastic feedstock and granulation temperature.

- Wan W, Zhang S, Huang H, Wu T. 2016. Occurrence and distribution of organophosphorus esters in soils and wheat plants in a plastic waste treatment area in China. *Environmental Pollution* 214, 349–353. https://doi.org/10.1016/j.envpol.2016.04.038 (with permission from Elsevier)

  This study for the first time reported the occurrence, distribution and concentrations of organophosphate esters (OPEs) in soils caused by plastic waste treatment, as well as their influence on OPE accumulation in wheat (Triticum aestivum L.). Eight OPEs were detected with the total concentrations of 38–1250 ng/g dry weight in the soils from the treatment sites, and tributoxyethyl phosphate and tri(2-chloroethyl) phosphate present as the dominant OPEs. There were similar distribution patterns of OPEs and significant correlations between the total OPE concentrations in the soils from the plastic waste treatment sites with those in the nearby farmlands ($P < 0.005$), indicating that plastic waste treatment caused the OPE contamination of farmland soils. The uptake and translocation of OPEs by wheat were determined, with OPEs of high hydrophobicity more easily taken up from soils and OPEs with low hydrophobicity more liable to be translocated acropetally.

- Some other relevant studies
  - Huang H, Wang D, Wan W, Wen B. 2017. Hexabromocyclododecanes in soils and plants from a plastic waste treatment area in North China: occurrence, diastereomer- and enantiomer-specific profiles, and metabolization. *Environmental Science and Pollution Research* 24, 21625–21635. https://doi.org/10.1007/s11356-017-9792-9
  - Asante KA, Amoyaw-Osei Y, Agusa T. 2019. E-waste recycling in Africa: risks and opportunities. *Current Opinion in Green and Sustainable Chemistry* 18, 109–117. https://doi.org/10.1016/j.cogsc.2019.04.001
  - Chakraborty P, Sampath S, Mukhopadhyay M, Selvaraj S, Bharat GK, Nizzetto L. 2019. Baseline investigation on plasticizers, bisphenol A, polycyclic aromatic hydrocarbons and heavy metals in the surface soil of the informal electronic waste recycling workshops and nearby open dumpsites in Indian metropolitan cities. *Environmental Pollution* 248, 1036–1045. https://doi.org/10.1016/j.envpol.2018.11.010

S2. Supporting Details on the Impacts of Chemical Additives on Waste-to-Energy

S2.1 Generation of large amounts of exhaust and bottom ashes containing significant levels of microplastics, hazardous additives and their thermal transformation products during incineration.

- Shen M, Hu T, Huang W, Song B, Qin M, Yi H, Zeng G, Zhang Y. 2021. Can incineration completely eliminate plastic wastes? An investigation of microplastics and heavy metals in the bottom ash and fly ash from an incineration plant. *Science of the Total Environment* 779, 146528. https://doi.org/10.1016/j.scitotenv.2021.146528 (with permission from Elsevier)
    - Incineration are generally considered to be the important elimination approaches of plastic wastes (Geyer et al., 2017; Yang et al., 2020a; Ye et al., 2020). Unfortunately, incomplete burning plastic fragments were found in the bottom ash, which means that microplastics may still exist in the bottom ash and can be transported to the environment via reuse or disposal (He et al., 2019).
    - The treatment and disposal of the bottom ash and fly ash of municipal solid waste incineration is an un-ignorable problem. The current results showed that there were a certain amount of microplastics and heavy metals in the fly ash and bottom ash generated from the incineration of municipal solid waste, polluting the surrounding soil environment. Macroplastics and microplastics were all found in the samples, and the content of microplastics in fly ash, bottom ash and soil was 23, 171, and 86 items/kg dw, respectively. The abundance of microplastics in bottom ash was significantly greater than that in around soils. The total proportion of (micro)plastics with different particle sizes in bottom ash, fly ash and soil were: < 0.5 (41.6%, 45.6%, 37.1%), 0.5–1 (27.2%, 30.6%, 25.9%), 1–2 (12.6%, 12.4%, 12.7%), 2–5 (10.2%, 10.2%, 15.7%), and > 5 mm (8.4%, 1.1%, 8.7%). The proportion of microplastics with smaller particle size was higher, and it was easier to diffuse and migrate into the surrounding environment.
- Matsukami H, Kajiwara N. 2019. Destruction behavior of short- and medium-chain chlorinated paraffins in solid waste at a pilot-scale incinerator. *Chemosphere* 230, 164–172. https://doi.org/10.1016/j.chemosphere.2019.05.048 (with permission from Elsevier)
    - Fig. 4 shows the flow rates of PCDD/DFs, dl-PCBs, HCB, and PeCB during the experimental incineration runs. The fact that the flow rates of these compounds in the samples of kiln exit gas exceeded the corresponding flow rates of the input materials indicated that thermal formation occurred during rotary kiln combustion at 840 °C. The respective order-of-magnitude increases of the flow rates of PCDD/DFs, dl-PCBs, HCB, and PeCB were 2.23, 3.43, 1.36, 2.97, and 3.50 for the SCCP-added RDF experimental incineration run, 2.31, 3.41, 1.44, 2.99, and 3.47 for the MCCP-added RDF experimental incineration run, and 1.8, 2.9, 0.52, 2.0, and 2.7 for the baseline RDF experimental incineration run. The fact that the rates of increase were higher in the SCCP-added and MCCP-added runs than in the baseline RDF incineration run suggested that SCCPs and MCCPs probably contributed to the unintentional formation of POPs in the rotary kiln primary combustion chamber.
    - The levels, flow rates and congener patterns of PCDD/DFs and dl-PCBs in each sample of environmental emission gas and ash obtained in this study were comparable to the previous study on incineration experiments using the same pilot-scale incinerator (Watanabe and Noma, 2010). The total concentrations of TEQs for PCDD/DFs in the samples of bottom and fly ash were lower than the Basel Convention provisional low POP content value of 15 ng-TEQ/g (UNEP, 2017b). On the other hand, the contamination levels of TEQs for PCDD/DFs and dl-PCBs in the ash samples obtained in this study were

still high compared to those in soils and eggs which may pose both environmental and human health risks (Weber et al., 2018). … Special attention should be paid to the presence of PCDD/DFs and dl-PCBs in the incinerator ash. Appropriate strategies and policies to prevent PCDD/DFs and dl-PCBs in the incinerator ash from entering the environment should be employed to reduce environmental and human health risks posed by PCDD/DFs and dl-PCBs in economically developing countries and regions towards the environmentally sound management of waste POPs.

- Yang H, Cheruiyot NK, Lin C, Wang L. 2022. Control of extreme brominated persistent organic pollutant emissions from start-ups of waste-to-energy incinerators. *Journal of Cleaner Production* 345, 131108. https://doi.org/10.1016/j.jclepro.2022.131108 (with permission from Elsevier)
    - Start-up processes of MSWIs [municipal solid waste incinerators] are characterized by unstable combustion conditions, which lead to extremely high emissions of products of incomplete combustion, including Cl-POPs (Cheruiyot et al., 2020; Neuer-Etscheidt et al., 2006; Tejima et al., 2007; Wang et al., 2007; Wyrzykowska-Ceradini et al., 2011a; Yang et al., 2015).
    - Wyrzykowska-Ceradini et al. (2011a) reported an average concentration of 50.1 pg WHO-TEQ Nm−3 for PBDD/Fs in the raw flue gas of a municipal waste combustor firing refuse-derived fuel during the start-up, which was 5-folds higher than that during steady-state conditions. The high concentrations during start-up were attributed to poor combustion conditions that led to PBDD/F formation from BFRs. The researchers also published a similar study on PBDE emissions and found that the average concentration during start-up was 38.8 ng Nm^{-3} (also 5-folds higher than during steady-state conditions) (Wyrzykowska-Ceradini et al., 2011b). The predominant congeners in the raw flue gas were those present in commercial deca-, octa-, and penta-BDEs, suggesting that these compounds desorb from the waste and get emitted without complete destruction.
    - This study found that start-up processes could contribute to at least 27%, 55%, and 2% PBDD/F, PBB, and PBDE emissions of an entire year of MSWI operations.

S2.2 Waste-to-fuel: in addition to contaminated exhaust and residues, the presence of many metals and halogenated chemicals may result in lower quality of the end fuel products, and thus, pose problems on the incinerators and other thermal facilities using the fuels.

- Chiang H, Lin K. 2014. Exhaust constituent emission factors of printed circuit board pyrolysis processes and its exhaust control. *Journal of Hazardous Materials* 264, 545–551. https://doi.org/10.1016/j.jhazmat.2013.10.049 (with permission from Elsevier)
    - Br content came from the flame-retardant additives, and some Si was determined from the epoxy resin and glass fibers in WPCB. The carbon, oxygen, and nitrogen content ranged from 263 to 103, 265 to 66, and 32 to 14 mg/g, respectively, corresponding to pyrolytic temperatures ranging from 200 to 500 °C. Bromine content did not significantly depend on the temperature, but high pyrolysis temperatures reduced the bromine content in residues, which indicates that bromine could be released to liquid or exhaust. Copper was the dominant metal in the pyrolytic residues (because the raw material is copper-clad laminates, and the copper could not be separated completely and removed from the

WPCB [waste printed circuit board] during crushing into fine particles), and its content decreased with the increase of temperature, indicating that most of the copper could be recycled after WPCB pyrolysis.

- Methanol, ethylacetate, acetone, dichloromethane, tetrachloromethane and acrylonitrile were the main species of oxygenated and chlorinated VOCs, and their emission factors were 333–1965, 589–2708 and 846–5269 μg/g, corresponding to pyrolysis temperatures of 300, 400 and 500 °C, respectively. Some chlorinated compounds were high, which may be attributed to the scrap PCB coming from the manufacturing site in the form of defective products. In addition, chlorinated compounds are used in the manufacturing process; therefore, some chloride could have remained on the WPCB (Table 1).
- High emission factor brominated compounds, i.e., bromoform, bromophenol, and dibromophenol, were determined at temperatures over 400 °C. Based on the exhaust compositions, some halogenated and oxygenated compounds, most of which are toxic, had high emission factors. Therefore, the exhaust gas has to be disposed of during the pyrolysis of WPCBs.

- Kusenberg M, Eschenbacher A, Djokic MR, Zayoud A, Ragaert K, De Meester S, Van Geem KM. 2022. Opportunities and challenges for the application of post-consumer plastic waste pyrolysis oils as steam cracker feedstocks: To decontaminate or not to decontaminate? *Waste Management* 138, 83–115. https://doi.org/10.1016/j.wasman.2021.11.009 (with permission from Elsevier)
  - Abstract
    - Thermochemical recycling of plastic waste to base chemicals via pyrolysis followed by a minimal amount of upgrading and steam cracking is expected to be the dominant chemical recycling technology in the coming decade. However, there are substantial safety and operational risks when using plastic waste pyrolysis oils instead of conventional fossil-based feedstocks. This is due to the fact that plastic waste pyrolysis oils contain a vast amount of contaminants which are the main drivers for corrosion, fouling and downstream catalyst poisoning in industrial steam cracking plants. Contaminants are therefore crucial to evaluate the steam cracking feasibility of these alternative feedstocks.
    - Indeed, current plastic waste pyrolysis oils exceed typical feedstock specifications for numerous known contaminants, e.g. nitrogen (~1650 vs. 100 ppm max.), oxygen (~1250 vs. 100 ppm max.), chlorine (~1460 vs. 3 ppm max.), iron (~33 vs. 0.001 ppm max.), sodium (~0.8 vs. 0.125 ppm max.) and calcium (~17 vs. 0.5 ppm max.). Pyrolysis oils produced from post-consumer plastic waste can only meet the current specifications set for industrial steam cracker feedstocks if they are upgraded, with hydrogen based technologies being the most effective, in combination with an effective pre-treatment of the plastic waste such as dehalogenation.
    - Moreover, steam crackers are reliant on a stable and predictable feedstock quality and quantity representing a challenge with plastic waste being largely influenced by consumer behavior, seasonal changes and local sorting

efficiencies. Nevertheless, with standardization of sorting plants this is expected to become less problematic in the coming decade.

  - More technical details can be found in the main text that is open-access.

- Yang X, Sun L, Xiang J, Hu S, Su S. 2013. Pyrolysis and dehalogenation of plastics from waste electrical and electronic equipment (WEEE): a review. *Waste Management* 33, 462–473. https://doi.org/10.1016/j.wasman.2012.07.025 (with permission from Elsevier)

  But, pyrolysis oils of WEEE plastics generally contain a large number of organic brominated compounds which apparently will hinder the reuse of them. Therefore it is necessary to upgrade the oil products or take dehalogenating measures during the pyrolysis process and directly obtain oils that are not contaminated by halogen. In fact, it is a crucial step, for the recycling of WEEE plastics, to remove organic halogen effectively and cheaply (Blazsó et al., 2002).

- Further studies on contaminated exhaust, residues and/or fuels

  - Wong S, Ngadi N, Abdullah TAT, Inuwa IM. 2015. Current state and future prospects of plastic waste as source of fuel: A review. *Renewable and Sustainable Energy Reviews* 50, 1167–1180. https://doi.org/10.1016/j.rser.2015.04.063.

  - Ma C, Yu J, Wang B, Song Z, Xiang J, Hu S, Su S, Sun L. 2016. Chemical recycling of brominated flame retarded plastics from e-waste for clean fuels production: a review. *Renewable and Sustainable Energy Reviews* 61, 433–450. https://doi.org/10.1016/j.rser.2016.04.020. – see Section 3.1.

  - Damodharan D, Rajesh Kumar B, Gopal K, De Poures MV, Sethuramasamyraja. 2019. Utilization of waste plastic oil in diesel engines: a review. *Reviews in Environmental Science and Bio/Technology* 18, 681–697. https://doi.org/10.1007/s11157-019-09516-x

  - Venturelli M, Falletta E, Pirola C, Ferrari F, Milani M, Montorsi L. 2022. Experimental evaluation of the pyrolysis of plastic residues and waste tires. *Applied Energy* 323, 119583. https://doi.org/10.1016/j.apenergy.2022.119583. – see Table 7.

  - Kusenberg M, Zayoud A, Roosen M, Thi H, Abbas-Abadi MS, Eschenbacher A, Kresovic U, De Meester S, Van Geem KM. 2022. A comprehensive experimental investigation of plastic waste pyrolysis oil quality and its dependence on the plastic waste composition. *Fuel Processing Technology* 227, 107090. https://doi.org/10.1016/j.fuprodc.2021.107090. – see Section 3.3

  - Rollinson A, Oladejo J. 2020. *Chemical Recycling: Status, Sustainability, and Environmental Impacts.* Global Alliance for Incinerator Alternatives. doi:10.46556/ONLS4535. https://www.no-burn.org/wp-content/uploads/2021/11/CR-Technical-Assessment_June-2020_for-printing-1.pdf

  - Rollinson AN. 2018. Fire, explosion and chemical toxicity hazards of gasification energy from waste. *Journal of Loss Prevention in the Process Industries* 54, 273–280. https://doi.org/10.1016/j.jlp.2018.04.010

- Rollinson AN, Oladejo JM. 2019. 'Patented blunderings', efficiency awareness, and self-sustainability claims in the pyrolysis energy from waste sector. *Resources, Conservation and Recycling* 141, 233–242. https://doi.org/10.1016/j.resconrec.2018.10.038
- Jahirul MI, Rasul MG, Schaller D, Khan MMK, Hasan MM, Hazrat MA. 2022. Transport fuel from waste plastics pyrolysis – a review on technologies, challenges and opportunities. *Energy Conversion and Management* 258, 115451. https://doi.org/10.1016/j.enconman.2022.115451. – Sections 6 and 10

S3. Supporting Details on the Impacts of Chemical Additives on Chemical Recycling

S3.1 Negative impacts of chemical additives on high-temperature processes such as using pyrolysis oils as steam cracker feedstocks and gasification

- Kusenberg M, Eschenbacher A, Djokic MR, Zayoud A, Ragaert K, De Meester S, Van Geem KM. 2022. Opportunities and challenges for the application of post-consumer plastic waste pyrolysis oils as steam cracker feedstocks: To decontaminate or not to decontaminate? *Waste Management* 138, 83–115. https://doi.org/10.1016/j.wasman.2021.11.009 (with permission from Elsevier)
    - Abstract
        - Thermochemical recycling of plastic waste to base chemicals via pyrolysis followed by a minimal amount of upgrading and steam cracking is expected to be the dominant chemical recycling technology in the coming decade. However, there are substantial safety and operational risks when using plastic waste pyrolysis oils instead of conventional fossil-based feedstocks. This is due to the fact that plastic waste pyrolysis oils contain a vast amount of contaminants which are the main drivers for corrosion, fouling and downstream catalyst poisoning in industrial steam cracking plants. Contaminants are therefore crucial to evaluate the steam cracking feasibility of these alternative feedstocks.
        - Indeed, current plastic waste pyrolysis oils exceed typical feedstock specifications for numerous known contaminants, e.g. nitrogen (~1650 vs. 100 ppm max.), oxygen (~1250 vs. 100 ppm max.), chlorine (~1460 vs. 3 ppm max.), iron (~33 vs. 0.001 ppm max.), sodium (~0.8 vs. 0.125 ppm max.) and calcium (~17 vs. 0.5 ppm max.). Pyrolysis oils produced from post-consumer plastic waste can only meet the current specifications set for industrial steam cracker feedstocks if they are upgraded, with hydrogen based technologies being the most effective, in combination with an effective pre-treatment of the plastic waste such as dehalogenation.
        - Moreover, steam crackers are reliant on a stable and predictable feedstock quality and quantity representing a challenge with plastic waste being largely influenced by consumer behavior, seasonal changes and local sorting efficiencies. Nevertheless, with standardization of sorting plants this is expected to become less problematic in the coming decade.

- More technical details can be found in the main text that is open-access, including 10th paragraph in the *Introduction* section, last paragraph of Section *3.3 Metal contaminant levels in liquid plastic waste pyrolysis products*, 2nd and 3rd paragraphs of Section *4.2.3 Halogens*, section *4.2.5 Phosphorus,* 2nd and 3rd paragraphs of Section 4.3 *Steam cracking feasibility of pyrolysis oils in terms of metals and inorganics*, Section *4.3.1 Aluminum*, Section *4.3.2 Antimony*, Section *4.3.3 Barium*, Section *4.3.4 Calcium*, Section *4.3.6 Copper*, Section *4.3.7 Iron*, Section *4.3.8 Potassium*, Section *4.3.9 Sodium*, Section *4.3.11 Silicon*, Section *4.3.12 Titanium*, Section *4.3.13 Zinc*, and 2nd and 3rd paragraphs of Section *6. Conclusion and outlook.*

- Kusenberg M, Roosen M, Zayoud A, Djokic MR, Thi HD, De Meester S, Ragaert K, Kresovic U, Van Geem KM. 2022. Assessing the feasibility of chemical recycling via steam cracking of untreated plastic waste pyrolysis oils: feedstock impurities, product yields and coke formation. *Waste Management* 141, 104–114. https://doi.org/10.1016/j.wasman.2022.01.033 (with permission from Elsevier)
  - In this work, steam cracking of two post-consumer plastic waste pyrolysis oils blended with fossil naphtha was performed in a continuous bench-scale unit without prior treatment. Product yields and radiant coil coke formation were benchmarked to fossil naphtha as an industrial feedstock. Additionally, the plastic waste pyrolysis oils were thoroughly characterized. Analyses included two dimensional gas chromatography coupled to a flame ionization detector for the detailed hydrocarbon composition as well as specific analyses for heteroatoms, halogens and metals. It was found that both pyrolysis oils are rich in olefins (~48 wt%) and that the main impurities are nitrogen, oxygen, chlorine, bromine, aluminum, calcium and sodium.
  - Steam cracking of the plastic waste derived feedstocks led to ethylene yields of ~23 wt% at a coil outlet temperature of 820 °C and ~28 wt% at 850 °C, exceeding the ethylene yield of pure naphtha at both conditions (~22 wt% and ~27 wt%, respectively). High amounts of heavy products were formed when steam cracking both pyrolysis oils, respectively. Furthermore, a substantial coking tendency was observed for the more contaminated pyrolysis oil, indicating that next to unsaturated hydrocarbons, contaminants are a strong driver for coke formation.
  - Both coke formation and fouling have been put in relation with heteroatoms and metal contaminants present in the post-consumer plastic waste pyrolysis oils. Consequently, using plastic waste derived feedstocks for industrial steam crackers poses opportunities and risks. On the one hand, these feedstocks are attractive as they help to close the material loop. On the other hand, use of these feedstocks may lead to operational issues because of increased coke formation and fouling. Therefore, the "unknowns" in plastic waste pyrolysis oils and their individual influence on industrial steam crackers must be further investigated.

- Cook E, Velis CA, Cottom JW. 2022. Scaling up resource recovery of plastics in the emergent circular economy to prevent plastic pollution: assessment of risks to health and safety in the Global South. *Waste Management & Research: The Journal for a Sustainable Circular Economy* https://doi.org/10.1177/0734242X221105415 (open-access with a CC BY license)

- Similarly to pyrolysis, gasification reactions involve the restriction of oxygen to allow decomposition of the polymers without complete combustion. Unlike pyrolysis, gasification takes place at higher temperatures (700–1200°C), and some oxygen is introduced into the process (Solis et al., 2020), resulting in partial oxidation of some hydrocarbons and atoms. Carbon monoxide (CO), hydrogen (H_2), CO_2, methane (CH_4) and nitrogen (N_2) are produced alongside some of the lower molecular weight hydrocarbons, such as ethane (C_2H_6) and ethylene (C_2H_4) (Ciuffi et al., 2020; Punkkinen et al., 2017). Collectively these are known as 'syngas'.
- Much heavier hydrocarbons are also produced resulting in a substance known as char alongside tarry substances. The tar is made up of a mixture of heterocyclic hydrocarbons such as pyridine and phenol, light aromatics such as benzene and toluene, polycyclic aromatic hydrocarbons such as naphthalene and heavier hydrocarbons that are not often characterised (Wolfesberger et al., 2009). This complex blend of substances is highly undesirable in the process as it quickly condenses, clogging and corroding pipework (Zeng et al., 2020). The char itself becomes contaminated by the tars, meaning it is unviable to clean, refine and utilise further (Benedetti et al., 2017; Lopez et al., 2018). The presence of these solids and their disposal continues to hinder the business case for gasification. Gasification of plastics produces less char compared to gasification of biomass or fibre (Sharuddin et al., 2016). However, the syngas itself tends to contain higher concentrations of char particulates; a key disadvantage to overcome when plastics are used as a feedstock (Lopez et al., 2018; Solis et al., 2020).
- The range of potentially hazardous substances produced by gasification and pyrolysis is not an inherent barrier to safe operation, and there are clearly engineering solutions to controlling process emissions, as detailed by Neuwahl et al. (2019). However, these controls are costly and require a high level of technical expertise to ensure that they are implemented and maintained to remain effective. Safe operation is not guaranteed anywhere in the world, and in countries that lack sufficiently well-resourced and effective enforcement and regulation, there is a risk that process emissions from gasification and pyrolysis may not be managed according to safe standards.

- Further studies
  - Rollinson AN. 2018. Fire, explosion and chemical toxicity hazards of gasification energy from waste. *Journal of Loss Prevention in the Process Industries* 54, 273–280. https://doi.org/10.1016/j.jlp.2018.04.010
  - Quicker P, Seitz M, Vogel J. 2022. Chemical recycling: a critical assessment of potential process approaches. *Waste Management & Research: The Journal for a Sustainable Circular Economy* 40(10), 1494–1504 https://doi.org/10.1177/0734242X221084044
  - Quicker, P. *Evaluation of Recent Developments Regarding Alternative Thermal Waste Treatment with a Focus on Depolymerisation Processes*; 2019. https://www.vivis.de/wp-content/uploads/WM9/2019_WM_359-370_Quicker.pdf

S4. Supporting Details on the Impacts of Chemical Additives on Bio-based Plastics

S4.1 Similar levels and complexity of chemicals found in bio-based plastics compared to petroleum-based plastics – leaching of (toxic) chemicals in observed in migration experiments

- Zimmermann L, Dombrowski A, Voelker C, Wagner M. 2020. Are bioplastics and plant-based materials safer than conventional plastics? In vitro toxicity and chemical composition. *Environmental International*, 106066. https://doi.org/10.1016/j.envint.2020.1060660 (open-access with CC-BY license)
    - Although the individual compounds will be specific to the material, conventional as well as bio-based and biodegradable plastics can contain all these chemical categories. Additives are particularly relevant for polymers extracted from natural resources, such as starch and cellulose, or from microorganisms, such as PLA, because of their limited physical properties, such as thermal resistance and barrier properties.
    - We tentatively identified a range of plausible compounds in bioplastics and plant-based materials. We found a number of plastic additives, including butanediyldihexadecanamide, ethylenebis(palmitamide), erucamide and Irganox 1076 as well as NIAS, including tetraoxacyclotetracosane-tetrone, a migrate from PE packaging (Sage et al., 2018) that is very similar to a NIAS found in biodegradable packaging (Canellas et al., 2015) and tris(2nonylphenyl) phosphate (in Bio-PE) which is a degradation product of the antioxidant tris(nonylphenyl) phosphite (TNPP) and has been detected in PE (Celiz et al., 2020).
- Zimmermann L, Bartosova Z, Braun K, Oehlmann J, Voelker C, Wagner M. 2021. Plastic Products Leach Chemicals That Induce In Vitro Toxicity under Realistic Use Conditions. *Environmental Science & Technology* 55, 17. https://doi.org/10.1021/acs.est.1c01103
    - By performing both extraction experiments, as well as migration experiments simulating realistic use conditions, Zimmermann and coworkers detected the presence and release of a wide range of chemicals, several of which induced *in vitro* toxicity, both from petroleum-based and from bio-based plastics (e.g., PLA).

S4.2 Chemical additives added to bio-based plastics based polylactic acid (PLA) to achieve durability for certain applications

- Tripathi N, Misra M, Mohanty AK. 2021. Durable Polylactic Acid (PLA)-Based Sustainable Engineered Blends and Biocomposites: Recent Developments, Challenges, and Opportunities. *ACS Engineering Au* 1, 7-38. https://doi.org/10.1021/acsengineeringau.1c00011
- Ainali NM, Kalaronis D, Evgenidou E, Kyzas GZ, Bobori DC, Kaloyianni M, Yang X, Bikaris DN, Lambropoulou DA. 2022. Do poly(lactic acid) microplastics instigate a threat? A perception for their dynamic towards environmental pollution and toxicity. *Science of The Total Environment* 832, 155014. https://doi.org/10.1016/j.scitotenv.2022.155014
    - As with petroleum-based plastics, the additive make-up of a bio-based plastic will depend on the desired application. Polylactic acid (PLA) is one of the most prominent bio-based plastics, used in a wide range of applications, including in biodegradable applications. However, the brittleness of PLA, its sensitivity to humidity and

susceptibility to weathering, requires the addition of fillers or chemical additives to enhance its durability for application requiring a higher longevity.

S4.3 The production of food-contact plastics based on high levels of plant fibers involves the use of several indispensable additives that may pose high risks to food safety.

- Bouma K, Wijk DKK, Sijm DTHM. Migration of Formaldehyde from 'Biobased' Bamboo/Melamine Cups: A Dutch Retail Survey. Chemosphere 2022, 292, 133439. https://doi.org/10.1016/j.chemosphere.2021.133439 (with permission from Elsevier)
    - Bamboo/melamine cups had become popular, due to their ecofriendly and sustainable image. Some cups are marketed as biobased and biodegradable. These cups are made from melamine-formaldehyde resin to which bamboo fibers or powder are added. They may release formaldehyde and melamine into the beverage. In 2019 the Netherlands Food and Consumer Product Safety Authority conducted a market study. 46 cups were sampled in duplicate and tested for formaldehyde migration. 88% of the cups complied with the migration limit of 15 mg/kg. Some cups showed very high migration values, over 200 mg/kg. There is a high exposure of formaldehyde from other sources (food, environment). When applying a realistic worst-case scenario of a daily intake of 0.4 L of beverage from the cups, the health based guidance value is exceeded at a migration of 19 mg/kg and higher. For some of the cups that had a high migration of formaldehyde, health risks may occur. For food-utensils for babies and young children, the exposure is higher due to their lower body weight. The SML does not provide sufficient protection for babies and young children. Bamboo is not authorized in the EU as additive for plastic food contact materials. Therefore the Netherlands, Belgium, Luxembourg and other EU member states have actively banned these products from the market. Finally, bamboo/melamine tableware would suggest having a 'green' image and would contribute to the goals of the circular economy to use biobased raw materials, but by using it in combination with melamine-formaldehyde resin may affect food safety.
- Zhang H, Weng Y. Safety Risks of Plant Fiber/Plastic Composites (PPCs) Intended for Food Contact: A Review of Potential Hazards and Risk Management Measures. Toxics 2021, 9 (12), 343. https://doi.org/10.3390/toxics9120343 (open-access with CC-BY license)
    - To date, excessive migration of hazardous substances (such as melamine) has been reported in some products made of PPCs, and the safety and applicability of PPCs as food contact materials need to be further studied.
    - The surface of plant fiber is rich in hydroxyl and carbonyl, which makes plant fiber hydrophilic [24]. However, synthetic resins are mostly nonpolar structures resulting in poor compatibility between the two phases when they are blended with plant fibers [26,33,34], manifested by peeling between two phases, material strength decrease, and poor processability [7]. To increase the compatibility and improve the performance of PPCs, it is usually necessary to introduce proper functional groups for the surface modification of plant fiber to reduce the hydrophilicity, or use additives such as plasticizers and compatibilizers in the compounding process [4,6].
    - Surface Modification of Plant Fiber.

Silane is a commonly used surface treatment agent. Cellulose can be treated with silanol aqueous solution, or silane coupling agents [47]. Olive husk flour [28], and a bamboo cellulose nanowhisker [47] treated with different silanes were all found to disperse more evenly in composites. Furthermore, the interfacial compatibility of PPCs was enhanced, and the mechanical properties and thermal stability properties were improved to varying degrees. However, an excessive amount of silanes would lead to its self-condensation reaction, which would cause insufficient silylation reaction and a lower grafting degree of the functional group [47].

Besides silane, other substances can also be used for the surface modification of plant fibers. Pyrrole can be oxidized and polymerized on the surface of bamboo fiber, and the resulting polypyrrole can improve the compatibility between bamboo fiber and PLA, and thus improve the mechanical properties and thermal stability of composites [48].

Alkali treatment is also a commonly used surface treatment method for plant fibers. Alkaline alkylation reaction occurs on the treated fiber surface, which is beneficial to blending with synthetic resins [7]. However, alkali treatment may reduce the inherent strength of plant fibers [47,49]. In a study, palm fiber (Macaíba) was first treated with maleic anhydride, sodium hydroxide or (3-methacryloxypropyl) trimethoxysilane (CAS: 2530-85-0), and then blended with polycaprolactone (PCL) [50]. The effect of this blend on mechanical properties of the composites was investigated, which showed that PPC with maleic anhydride-modified fiber had the best mechanical property, while sodium hydroxide had the worst modification effect.

- Compatibilizer

Compatibilizers are often used to improve the properties of PPCs. Maleic anhydride, as a common reactive compatibilizer, can undergo esterification reaction with hydroxyl groups on the fiber surface, thus enhancing adhesion power between plant fiber and synthetic resin and improving the mechanical property of materials [7,51]. Compared with common PLA, adding 0.3% maleic-anhydride-grafted-PLA as a compatibilizer can improve the mechanical properties and waterproof performance of wood fiber/PLA composites [52]. Similar results were reported for the composite of bamboo fiber and PP using maleic-anhydride-grafted-PP as compatibilizer [22], and corn straw powder/low-density polyethylene (LDPE) composite compatibilized by maleic-anhydride-grafted-PE [53]. Lignin has also been reported as a coupling agent to increase the compatibility between plant fiber and plastic matrix, thus improving the mechanical properties of composites [42].

- Other Additives

Additives commonly used in PPCs also include plasticizers, water and oil repellent, filling agent, nucleator, etc. [54]. Commonly used plasticizers include glycerol, ethylene glycol, urea, aliphatic acid, sugar alcohol, etc. [7]. Coffee silverskin/PHBV composites plasticized by acetyl tributyl citrate (ATBC) showed a better processability [39]. In the study of additives used in tableware made of ramie sticks, 3% liquid paraffin was found to bring a better waterproof effect, while lime carbonate, talcum powder, and white clay as composite filling agents could make the tableware have the strongest oil resistance [55]. Nano-silica is a common nucleator, which was reported to enhance mechanical

properties, water resistance, and thermal stability of bamboo fiber/PLA composites when added up to 1.5% [56].

- The safety risks introduced by synthetic resins mainly come from residual monomers, polymer decomposition products, oligomers, etc. MF resin, as a commonly used thermoset material [65], has been blended with plant fibers to produce tableware in many applications. However, MF resin will decompose under acidic conditions or high temperatures, resulting in the migration of melamine and formaldehyde [66]. Formaldehyde residues were tested in food contact materials made of various fiber/MF composites [57]. The migration of 25 volatile and semi-volatile substances, and 12 non-volatile substances have been found in bamboo/MF food contact materials, of which non-volatile substances were mainly melamine and its derivatives [8]. Federal Office of Consumer Protection and Food Safety of Germany (BMEL) randomly inspected 56 kinds of products on the German market and found that 11% of bamboo powder or corn starch tableware samples had excessive formaldehyde migration and 25% of samples had excessive melamine migration [67].

- Additives, with relatively low molecular weight and high reactivity, are easier to migrate and may have higher safety risks. Long-term exposure of maleic anhydride, which is commonly used in PPCs, will cause certain damage to the respiratory system, digestive system, and kidney [71,72]. Many countries and regions have also set a migration limit for this substance [73,74]. The migration of phthalates as a plasticizer, benzophenonone (BP) and 4-methylbenzophenonone (4MBP), which may be photoinitiators from photo-cured printing inks or adhesives, were also found in plant fiber-based materials [67,75].
- In addition, the persistent organic contaminants perfluorooctane sulfonate (PFOS) and perfluorooctanoic acid (PFOA) that are refractory with long half-lives and have accumulation effects in organisms, can be used in plant fiber-based materials as surfactants for water-proof and oil-proof functions [76]. Relevant studies have shown that such substances may have reproductive and developmental toxicity and are related to cancer and thyroid diseases [77,78]. Thus, the possibility of perfluorinated or polyfluorinated substances migration should also be considered to avoid associated risks.

S5. Supporting Details on the Impacts of Chemical Additives on Biodegradable Plastics

S5.1 Biodegradable plastics (petroleum- or biobased) only fully biodegrade (i.e., mineralize) in industrial composting facilities, but partial disintegration and/or slow degradation in the natural environment may lead to formation of microplastics and/or release of chemical additives.

- Kubowicz S, Booth AM. 2017. Biodegradability of Plastics: Challenges and Misconceptions. *Environmental Science & Technology* 51, 12058-12060. https://doi.org/10.1021/acs.est.7b04051

- Ferreira-Filipe DA, Paco A, Duarte AC, Rocha-Santos T, Patricio Silva AL. 2021. Are Biobased Plastics Green Alternatives?—A Critical Review. *International Journal of Environmental Research and Public Health* 18, 7729. https://doi.org/10.3390/ijerph18157729 (open-access with CC-BY license)

- Hence, carelessly branding biobased plastics as green plastics might instill the wrong ideas in the minds of the consumers—the consequences of discarding these plastics, biodegradable or not, might be unintentionally ignored by the consumer lulled by the false sense of security given off by that green branding [75]; even certified biodegradable plastics are so only under specific co nditions (e.g., in industrial composting facilities/bioreactors).

- Rujnić-Sokele M, Pilipović A. 2017. Challenges and opportunities of biodegradable plastics: A mini review. *Waste Management & Research* 35 (2), 132-140. https://doi.org/10.1177/0734242X16683272
    - The process of biodegradation differs strongly between different environments (mostly dependent on temperature and presence of biodegrading microorganisms), with the most aggressive environment typically being compost, then soil, followed by fresh and marine water and finally landfill. Most standards (like EN 13432) for compostable or biodegradable plastics only refer to industrial compositing and are not applicable to natural environments.

- Rosenboom J-G, Langer R, Traverso G. 2022. Bioplastics for a circular economy. *Nature Reviews Materials* 7, 117–137. https://doi.org/10.1038/s41578-021-00407-8
    - Of note is also the special case of oxo-degradable plastics, which are formulated with additives that render otherwise persistent plastics degradable, but leave behind poorly degradable microplastic fragments (their use is therefore restricted in many countries).

S6. Supporting Details on the Impacts of Chemical Additives on Durable Plastics

S6.1 Migration, transformation and adsorption of chemicals from plastic water bottles during dishwashing.

- Tisler S and Christensen JH 2022. Non-target screening for the identification of migrating compounds from reusable plastic bottles into drinking water. *Journal of Hazardous Materials* 429, 128331. https://doi.org/10.1016/j.jhazmat.2022.128331 (open access with CC-BY license)
    - In this study, we investigated the migration of FCMs from plastic bottles into drinking (tap) water over 24h at room temperature. We detected > 400 plastic related compounds as well as > 3500 dishwasher related compounds. The study shows the importance of considering special cleaning steps for plastic bottles. Generally, dishwasher related compounds were found to adsorb more to plastic than glass, and especially the more non-polar compounds were difficult to remove even by additional water flushing afterwards. Furthermore, the dishwashing process enhanced the migration of plasticizers, antioxidants, and photoinitiators into the drinking water. Therefore, the highest predicted toxic hazard was calculated for the used plastic bottles, that had been refilled directly after the dishwasher, without further flushing. However, 15 out of the 20 highest peaks in the new bottles were assigned to oligomers of plasticizers, as well as aromatic amines which could potentially originate from slip agents or antioxidants. These compounds showed continuous leaching, even after flushing. To our knowledge, no toxicity data are available for these compounds. The identification of DEET in the plastic bottles also raised

the question whether the ubiquitous detection of DEET from its use as an insect repellant is correct, or if it has another source.

S6.2 Migration of additives from plastics accelerates product embrittlement and reduces durability.

- Hale RC, King AE, Ramirez JM, La Guardia M, Nidel C. 2022. Durable Plastic Goods: A Source of Microplastics and Chemical Additives in the Built and Natural Environments. *Environmental Science & Technology Letters* 9(10), 798–807. https://doi.org/10.1021/acs.estlett.2c00417
    - Additives are often essential to maintain plastic product performance (and safety). Through migration of additives out of durable plastics over time embrittlement and failure of plastic products is accelerated. Migration influenced by ambient chemical and physical conditions (and therefore depends on the type and location of application).

S6.3 Increased stability of plastics through the protective function of specific chemical additives can have unwanted side-effects.

- Tian Z et al. 2020. A ubiquitous tire rubber–derived chemical induces acute mortality in coho salmon. *Science* 371, 185-189. https://doi.org/10.1126/science.abd6951
    - The tire rubber additive N-(1,3-dimethylbutyl)-N′-phenyl-p-phenylenediamine (6PPD) is designed to protect the tire rubber against ozone-mediated oxidation. However, 6PPD transforms to 6PPD-quinone (and related transformation products) upon deploying its protective antioxidative function, a chemical that is now known to be highly toxic to aquatic species, such as coho salmon.
